# Th2-biased immune responses to body migrating *Ascaris* larvae in primary infection are associated with pathology but not protection

**DOI:** 10.1038/s41598-024-65281-0

**Published:** 2024-06-28

**Authors:** Luis E. Elizalde-Velázquez, Josephine Schlosser-Brandenburg, Alexandra Laubschat, Larissa Oser, Arkadi Kundik, Joshua Adjah, Saskia Groenhagen, Anja A. Kühl, Sebastian Rausch, Susanne Hartmann

**Affiliations:** 1 Institute of Immunology, Centre for Infection Medicine, Department of Veterinary Medicine, Freie Universität Berlin, Berlin, Germany; 2 Charité - Universitätsmedizin Berlin, Corporate Member of Freie Universität Berlin and Humboldt Universität zu Berlin, iPATH.Berlin, Berlin, Germany

**Keywords:** Parasitic infection, Innate immunity, Parasitic infection, Mucosal immunology

## Abstract

Helminth infections lead to an overdispersion of the parasites in humans as well as in animals. We asked whether early immune responses against migrating *Ascaris* larvae are responsible for the unequal distribution of worms in natural host populations and thus investigated a susceptible versus a resistant mouse strain. In mice, the roundworm larvae develop until the lung stage and thus early anti-*Ascaris* immune responses against the migrating larvae in the liver and lung can be deciphered. Our data show that susceptible C57BL/6 mice respond to *Ascaris* larval migration significantly stronger compared to resistant CBA mice and the anti-parasite reactivity is associated with pathology. Increased eosinophil recruitment was detected in the liver and lungs, but also in the spleen and peritoneal cavity of susceptible mice on day 8 post infection compared to resistant mice. In serum, eosinophil peroxidase levels were significantly higher only in the susceptible mice, indicating functional activity of the recruited eosinophils. This effect was associated with an increased IL-5/IL-13 production by innate lymphoid cells and CD4^+^ T cells and a pronounced type 2 macrophage polarization in the lungs of susceptible mice. Furthermore, a comparison of wildtype BALB/c and eosinophil-deficient dblGATA-1 BALB/c mice showed that eosinophils were not essential for the early control of migrating *Ascaris* larvae. In conclusion, in primary infection, a strong local and systemic type 2 immune response during hepato-tracheal helminth larval migration is associated with pathology rather than protection.

## Introduction

Infections with soil-transmitted helminths (STH) represent a considerable public health concern worldwide, affecting humans and life stock alike^1–3^. A recent meta-analysis of global *Ascaris* prevalence has highlighted a prevalence estimate of over 0.7 billion individuals being infected worldwide^4^. However, the limited available data on infection intensity indicates that the true burden may be higher. There is a considerable degree of regional variation in *Ascaris* prevalence, with a low prevalence (less than 4%) observed in countries in East Asia and West Asia, and a high prevalence (greater than 20%) observed in some countries in Oceania, Southeast Asia, the Latin American and Caribbean region, sub-Saharan Africa and South Asia^4^. In 2019, the global disease burden of ascariasis was estimated to be 754,000 disability-adjusted life years (DALYs) (95% UI 479,000-1,140,000), with a total of 2090 deaths (1640–2640). Children aged 1–14 years were found to be the most affected (https://www.healthdata.org/results/gbd_summaries/2019/ascariasis-level-4-cause).

While rarely fatal, the chronic medium- to high-intensity infections are associated with growth retardation and cognitive impairment in children, as well as with anaemia^2,3,5,6^. A small minority of individuals harbors the majority of worms and this overdispersion is linked with the degree of acute and chronic morbidity^7^. To date, the factors responsible for the overdispersion of parasite burdens in host populations remain insufficiently understood. Some studies suggest that variation in the host genotype and behavior as well as in environmental factors are involved^8,9^. Differential innate immune responses elicited against the larval stages of the parasites may constitute another major determinant, which so far remained understudied in natural hosts^10^.

In *Ascariasis*, the complex life cycle affecting multiple organ systems has hindered the definition of critical immune parameters associated with parasite overdispersion. After hatching from ingested eggs in the host gut, the larvae undergo extensive tissue migration via liver and lung back to the small intestine, followed by the expulsion of the majority of worms^11^. Given the limitations of human studies, the use of an animal model for the study of ascariasis has proven to be an effective approach, particularly for elucidating the mechanisms of the immune response and pathophysiology. For practical reasons, investigations of the associated immune responses are challenging in the pig, the natural host of *A. suum* infection^12,13^. Hence, mice were established as an artificial host system, facilitating the investigation of the immune responses to the tissue-migratory phase of *Ascaris* infection^14,15^.

The mouse model does not permit the development of *Ascaris* to the fourth larval stage and the following transition to the adult stage. However, the larval stages pass through the liver and lungs, which enables the analysis of the early anti-*Ascaris* immune response against the migrating larvae in these organs^12,16,17^. While the hepatic immune responses during larval migration in mice have yet to be fully elucidated, extensive research has already been conducted on the pulmonary immune response. Primary infections with *Ascaris* spp. in mice result in the development of a local inflammatory type 2 response in the lungs. This response is characterized by an early production of IL-5, followed by elevated levels of IL-4, IL-6, IL-33, eotaxin, CCL2 and CXCL10^18–20^. The inflammatory influx of cells into both the lung parenchyma and the bronchoalveolar fluid is initially dominated by neutrophils and eosinophils, followed by an accumulation of mononuclear cells that eventually differentiate into M2 macrophages^18,20^. During the pulmonary phase, the lesions caused by larval migration and excessive immune responses contribute to tissue remodeling, which is characterized by fibrosis and pulmonary dysfunction^20^. A recent study further demonstrated that eosinophils play an important role in regulating IgA production in mucosal sites, tissue inflammation, and remodeling, as well as controlling parasite burden during experimental ascariasis in mice^21^. Nevertheless, the precise mechanisms underlying the regulation of parasite load in primary *Ascaris* infection remain poorly understood. Previous studies investigating the susceptibility of various mouse strains to primary *Ascaris* infection have served as a foundation for research into these processes. Based on the numbers of third stage *Ascaris* larvae (L3) found in the lungs, C57BL/6 and CBA mice are considered susceptible and resistant, respectively^22,23^. The aforementioned murine model of susceptibility and resistance to primary *Ascaris* infection demonstrated that the inflammatory response in the tissue plays an integral role in regulating the parasite load, as evidenced by studies conducted by Lewis et al. and Dold et al.^22,24^. Proteomic analyses of the liver tissue of CBA/Ca and susceptible C57BL/6J mice infected with *A. suum* demonstrated relevant intrinsic differences between the two strains. These included oxidative phosphorylation and reactive oxygen species production, which may be involved in resistance mechanisms^17^. However, despite a number of studies in mouse models attempting to decipher early anti-*Ascaris* immune mechanisms that may be crucial for the hosts’ susceptibility to primary infection, the cellular immune signatures in both transmigrated organs, the liver and lung, have only been studied to a limited extent.

Consequently, we conducted a comparative study to examine the cellular immune responses elicited during the early migration of *A. suum* larvae through the liver and lungs of resistant CBA and susceptible C57BL/6 mice. Furthermore, an eosinophil-deficient mouse strain was included in the study to gain further insights into the underlying mechanisms of overdispersion.

## Results

### Higher numbers of *Ascaris* larvae are linked with more extensive liver and lung pathology in susceptible compared to resistant mice

**Figure 1:**
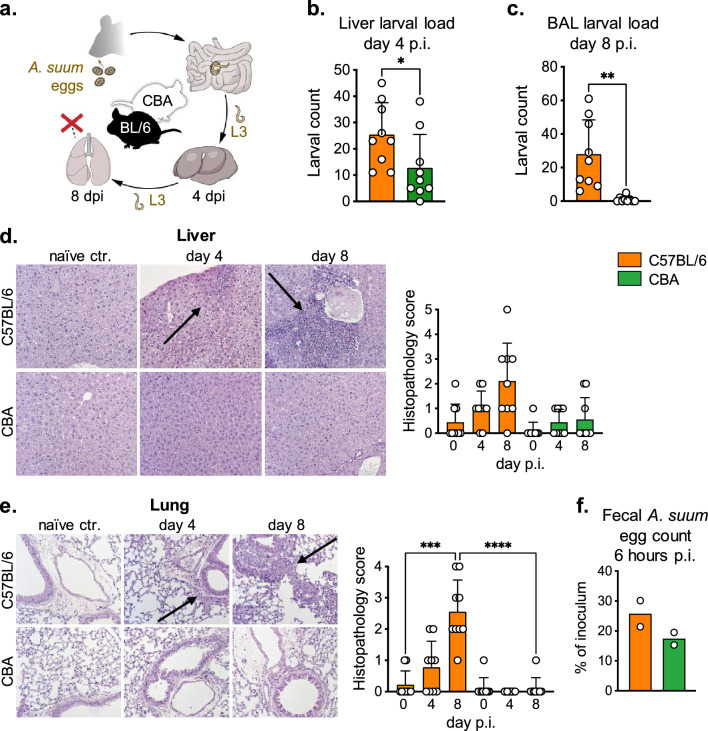
Increased susceptibility to *A. suum* larval migration leads to enhanced hepatic and pulmonary inflammation. (**a**) *Ascaris suum* life stages in the mouse model. (**b**, **c**) Larval loads in liver and bronchoalveolar lavage (BAL). (**d**, **e**) Hematoxylin and eosin stained liver and lung sections at day 4 and 8 p.i. Arrows indicate the cellular infiltration. Bar graphs compile 3 independent experiments. (**f**) Percentages of the infective *A*scaris egg inoculum released with feces within 6 h p.i. Data shown are pooled from 3 independent experiments with n = 3 animals per time point. p < 0.05 *; p < 0.01 **; p < 0.001 ***; p < 0.0001 ****.

To determine the degree of inflammation occurring in hepatic and pulmonary tissue during *Ascaris* L3 stage larvae (L3) hepato-tracheal migration on day 4 and day 8 post infection (p.i.) (Fig. 1a), we infected two mouse strains described as susceptible (C57BL/6) and resistant (CBA) to primary *A. suum* infection^22,23^. 2500 embryonated *A. suum* eggs were used to infect both mouse strains.

Quantifying the larval load of both mouse strains at the two time points reflecting the liver and lung stage of infection showed that C57BL/6 mice had significantly higher numbers of *Ascaris* L3 in the liver at day 4 p.i. compared to CBA mice (Fig. 1b). This difference became even clearer in the lung stage of infection. On day 8 p.i., the bronchoalveolar lavage (BAL) samples of all C57BL/6 mice comprised numerous *Ascaris* larvae, contrasting with the absence of larvae from most of the CBA mice-derived BAL samples (Fig. 1c). Thus, our data confirm earlier studies reporting a higher resistance of CBA mice compared to C57BL/6 mice against migrating *Ascaris* L3 during primary infection^22,23^.

Histological examination of liver and lung sections of both mouse strains demonstrated a trend of increased liver lesions in susceptible C57BL/6 mice at day 4 and 8 p.i. as compared to resistant CBA mice (Fig. 1d). In accordance with the low number of larvae recovered from the lung of CBA mice, lung pathology was negligible in this strain at day 8 p.i. and significantly different from the susceptible strain which showed clear lung lesions (Fig. 1e). Hence, C57BL/6 mice were more permissive to larval migration through the liver and lung. The higher larval burdens seen in both organs of C57BL/6 mice were associated with a stronger recruitment of inflammatory cells, resulting in increased pathology as compared to the more resistant CBA strain.

To assess whether equal amounts of larvae hatched in the two mouse strains, we determined the number of intact embryonated eggs passed with the feces within 6 h p.i.. As shown in Fig. 1f, two groups of C57BL/6 mice from independent experiments deposited about 26% (21.4 and 30.1%) of the applied infective dose within 6 h after inoculation, while the corresponding CBA groups passed about 17% (15.3 and 19.5%) of the eggs with the feces. The comparable percentages of infectious inoculum released by the two mouse strains suggest that susceptibility and resistance to *Ascaris* infection are not due to barrier differences, but rather to the nature of immune responses elicited by the susceptible and resistant mouse strains during *Ascaris* hepato-tracheal migration.

### *A. suum *larval migration is associated with local and systemic eosinophilia

**Figure 2:**
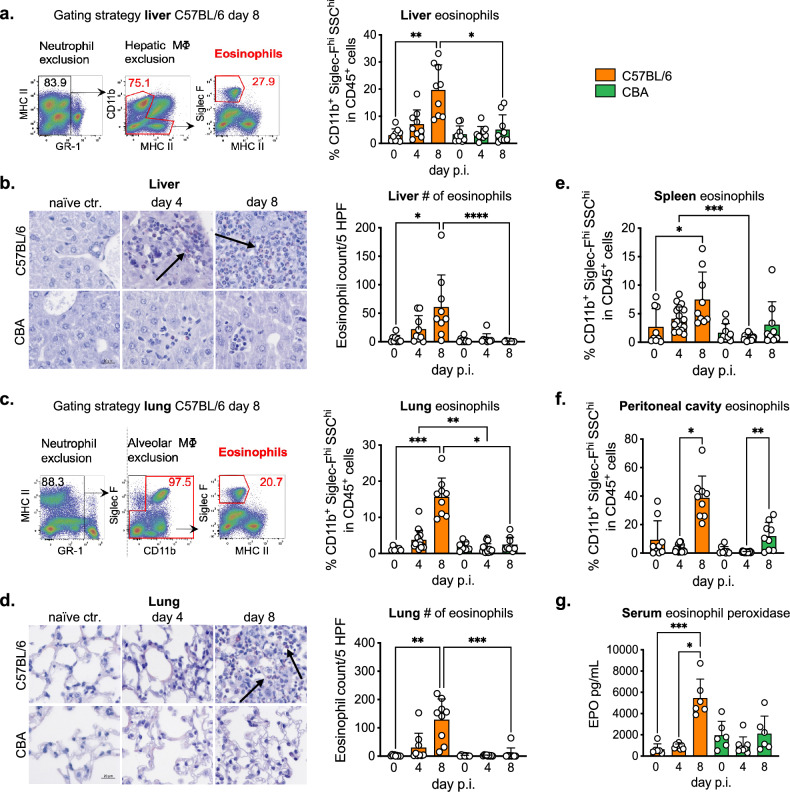
Susceptible C57BL/6 mice presents local and systemic eosinophilic infiltration and degranulation during larval migration. (**a**) Exemplary gating strategy used to identify eosinophils (Gr-1^-^Siglec-F^+^MHCII^-^) in liver tissue and frequencies of hepatic eosinophils. (**b**) Liver sections, eosinophils depicted with black arrows and numbers of eosinophils in the liver. (**c**) Exemplary gating strategy used to identify eosinophils (Gr-1^-^CD11b^+^Siglec F^+^) in lung tissue and frequencies of eosinophils in pulmonary CD45^+^ cells. (**d**) Lung sections and numbers of eosinophils in the lungs. (**e**, **f**) Frequencies of spleen eosinophils and peritoneal cavity eosinophils. (**g**) Eosinophil Peroxidase (EPO) levels detected in serum. Each symbol in bar plots represents an individual mouse. Data are pooled from 3 independent experiments with n = 3 to 6 animals per time point. p < 0.05 *; p < 0.01 **; p < 0.001 ***; p < 0.0001 ****.

The invasion of host tissue by the larval stages of parasitic helminths results in the rapid recruitment of eosinophils to the site of infection^25^ and eosinophils were reported to be important for the control of migrating *Strongyloides ratti* larvae^26^. We therefore determined the frequencies of eosinophils in the liver and lungs of both mouse strains via flow cytometry (Fig. 2a,c). Comparing day 4 p.i. to naïve controls, we detected similar frequencies of Siglec-F^+^ eosinophils in liver-derived CD45^+^ cells for both mouse strains (Fig. 2a). However, at day 8 p.i., C57BL/6 mice displayed a highly significant rise in hepatic eosinophils, whereas eosinophil frequencies remained low in resistant CBA mice (Fig. 2a). In trend, this pattern was reflected in the quantification of Sirius red stained eosinophils in histological sections of liver tissue (Fig. 2b). A similar difference was seen when quantifying the eosinophil responses in the lungs of the two mouse strains. C57BL/6 mice displayed a significant accumulation of eosinophils in lung tissue and BAL fluid at day 8 p.i., which contrasted with the unaltered eosinophil counts determined in infected CBA mice via flow cytometry (Fig. 2c) as well as in histological examinations of the lung tissue (Fig. 2d).

To see whether the differential resistance to infection might be associated with the degree of type 2 inflammation triggered by *Ascaris* larval migration at the systemic level, we analyzed the eosinophil responses based on peritoneal exudate in the peritoneal cavity (PEC) and spleen of both mouse strains. However, similar to the pattern seen in the organs directly affected by larval migration, a significant increase of eosinophils was seen in spleen and peritoneal cavity of the more susceptible C57BL/6 strain compared to resistant CBA mice at day 4 and 8 p.i. (Fig. 2e,f). Of note, the levels of eosinophil peroxidase (EPO) were also clearly increased in serum of C57BL/6 mice at day 8 p.i. and exceeded the overall amounts determined in serum of infected CBA mice (Fig. 2g). Hence, the more susceptible mouse strain developed a stronger local and systemic innate effector cell response to *Ascaris* infection, and the elevated serum EPO levels indicated that these cells were also functionally active in terms of the release of cytotoxic effector molecules.

### Strong type 2 cytokine production by ILC2 and CD4^+^ T cells along with pulmonary M2 polarization in susceptible mice

**Figure 3:**
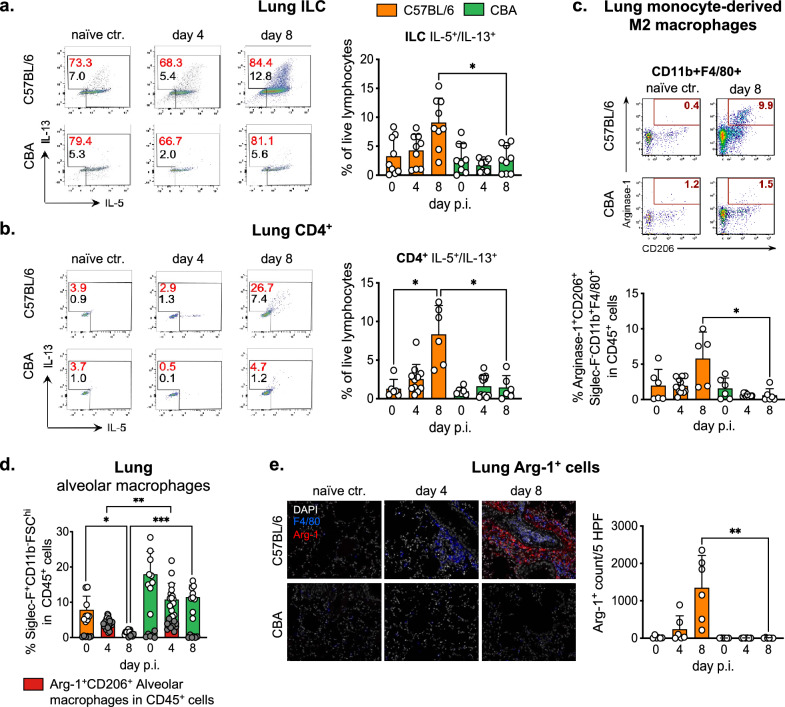
Susceptible C57BL/6 mice present local activation of type 2 response and pulmonary M2 polarization during larval migration. (**a**, **b**) Frequencies of lung IL-5^+^/IL-13^+^ innate lymphoid cells (ILC) and CD4^+^ T cells. The percentage of cytokine positive ILC and CD4^+^ T cells is reported by red numbers. Black numbers report the frequencies of cytokine producing cells in live lymphocytes. (**c**) Exemplary gating of arginase 1 (Arg-1) and CD206 expression by CD11b^+^F4/80^+^Siglec-F^-^ monocyte-derived macrophages from the lung tissue. Bar graph reports the frequencies of lung monocyte-derived M2 macrophages in pulmonary CD45^+^ live cells. (**d**) Superimposed bar graphs depicting the frequencies of Arg-1^+^CD206^+^ alveolar macrophages (red bar graphs and grey dots) within the total population of lung alveolar macrophages (white dots). (**e**) Lung sections depicting the merged stains with DAPI (white), F4/80 (blue) and Arg-1 (red) and number of arginase 1 positive cells (Arg-1^+^) per 5 high power fields (HPF) in lung tissue. Data shown are pooled from 2 to 3 independent experiments with n = 3 to 6 animals per time point. p < 0.05 *; p < 0.01 **; p < 0.001 ***.

To test whether the distinct eosinophil responses of the two mouse strains correlated with the production of type 2 cytokines, we determined IL-5 and IL-13 responses by innate lymphoid cells (ILC) and CD4^+^ T cells in liver (Supplementary Fig. 1) and lungs (Fig. 3a,b) of the two mouse strains. In parallel, we surveyed the extent of M2 polarization based on the expression of arginase 1 (Arg-1) and CD206 by liver-resident Kupffer cells (Tim-4^bright^) and monocyte-derived macrophages (Tim-4^dim^)^27^. While a trend for increased frequencies of ILC2 along the course of infection did not reach significance in the liver of C57BL/6 mice (Supplementary Fig. 1b), a significant accumulation of Th2 cells was seen in the liver of susceptible C57BL/6 mice but not in the resistant CBA strain at day 8 p.i. (Supplementary Fig. 1c). The clearly higher Th2 responses seen in the liver of C57BL/6 mice was accompanied by a significant increase in the numbers of monocyte-derived Tim-4^dim^ macrophages in liver tissue of the more susceptible mouse strain (Supplementary Fig. 1d). Interestingly, monocyte-derived macrophages also appeared to be primarily accountable for the modestly stronger M2 responses seen in the liver of some C57BL/6 mice (Supplementary Fig. 1e), whereas M2 marker expression by TIM-4^hi^ liver-resident Kupffer cells was similar at all time points and between the mouse strains.

In accordance with the higher larval counts and stronger eosinophil responses seen in the lungs of C57BL/6 mice, these mice also displayed a significant increase in IL-5/IL-13 producing ILC2 as well Th2 cells at day 8 p.i. in the lung compared to resistant CBA mice (Fig. 3a,b). The stronger ILC2 and Th2 responses were further associated with the significantly more pronounced M2 polarization evident in the elevated proportion of Arg-1/CD206 double-expressing cells within CD11b^+^F4/80^+^Siglec-F^-^ macrophages isolated from lung tissue and BAL of C57BL/6 mice at day 8 post infection (Fig. 3c and data not shown). Reminiscent of the situation in the liver at day 8 post infection, also the lung M2 response appeared to be dominated by monocyte-derived macrophages, whereas tissue-resident Siglec-F^+^ alveolar macrophages present in lung tissue and BAL fluid did not contribute to Arg-1 expression in susceptible C57BL/6 or resistant CBA mice at day 8 p.i. (Fig. 3d).

Immunofluorescent staining confirmed the elevated Arg-1 expression to be restricted to the lung of *Ascaris* infected C57BL/6 mice (Fig. 3e). Of note, the average Arg-1^+^ cell count determined in the lung sections exceeded the mean numbers of F4/80^+^ cells (Arg-1^+^ and Arg-1^-^) by the factor of 1.2 (+ /- 0.6) (Fig. 3e). Thus, our data indicate that the M2 phenotype was largely confined to recently attracted, monocyte-derived F4/80 low/negative macrophages as the number of F4/80^+^Arg-1^+^ cells detected in lung tissue constituted 9.4% (+ /- 11.1%) of the total Arg-1^+^ cells. Tissue-resident alveolar macrophages (typically F4/80^dim^ and Siglec-F^+^^28^) poorly contributed to Arg-1 expression in the context of *Ascaris* lung stage infection as previously shown in Fig. 3d.

### The lack of eosinophils does not change susceptibility to infection

The data shown so far implied that the genetical resistance of CBA mice operates against the short-term infection with *Ascaris* larvae in the absence of clearly discernable type 2 immune activity. We therefore asked whether the early innate type 2 response seen in the susceptible mouse strain at least contributed to the control of *Ascaris* larvae. Eosinophil responses were previously reported to be associated with protection against recurrent *Ascaris* infection in pigs^29^, as well as with immune protection against other tissue-migratory nematodes^26,30^. We therefore focused on the impact of the robust local and systemic eosinophilia seen in C57BL/6 mice. Although dblGATA-1 mice and other eosinophil-deficient mouse strains are available on the C57BL/6 background, we chose the dblGATA-1 model on the BALB/c genetic background for several reasons. First, earlier studies demonstrated the intermediate resistance of the BALB/c strain against *Ascaris* infection, ranging between the highly susceptible C57BL/6 and highly resistant CBA strains^23^, leaving room for the detection of negative as well as positive effects of eosinophil deficiency. Second, BALB/c mice are considered more Th2 prone compared to C57BL/6^31^, potentially offering a model system reporting effective type-2 mediated control of early stage *Ascaris* infections. We therefore reasoned that depriving this strain of modest protective effects possibly conveyed by the activity of eosinophils might result in L3 counts intolerable to the host and in severe organ damage during infection.

**Figure 4:**
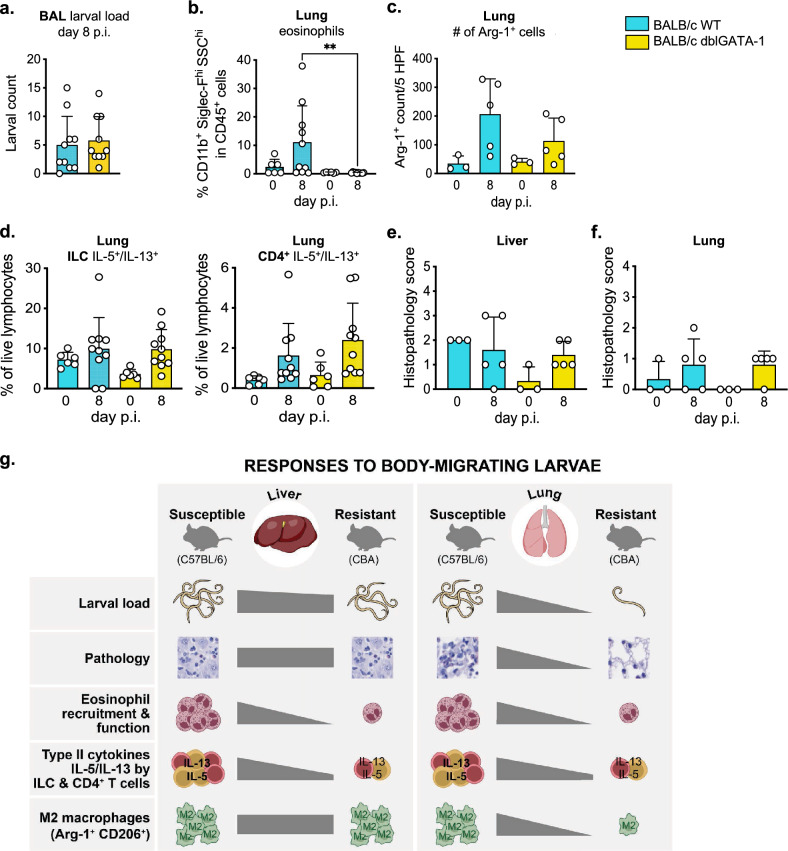
Lack of eosinophils does not alter the course of *A. suum* primary infection. (**a**) Larval load in bronchoalveolar lavage (BAL) (**b**) Frequencies of eosinophils in lung cells determined by flow cytometry. (**c**) Number of arginase 1 positive cells (Arg-1^+^ cells) in lung. (**d**) Frequencies of lung IL-5^+^/IL-13^+^ innate lymphoid cells (ILCs) and CD4^+^ T cells. (**e**) Liver and (**f**) lung histopathology scoring. Pooled data from 2 independent experiments with n = 3 to 5 animals per time point. p < 0.01 **. (**g**) Graphic summarizing the responses to body-migrating *A. suum* larvae in liver and lung in the susceptible and resistant mouse strain.

We hence compared the course of *Ascaris* infection in eosinophil deficient dblGATA-1 mice^32^ and the respective BALB/c wild type (WT) controls. The larval counts isolated from the lung of BALB/c WT and age- and sex-matched dblGATA-1 mice at day 8 p.i. were highly similar (Fig. 4a), indicating that the lack of eosinophils did not alter susceptibility to *Ascaris* infection. Contrasting the nearly complete prevention of lung stage infection seen earlier in CBA mice, low numbers of L3 were retrieved from the lung of all but one BALB/c mouse at day 8 p.i. As expected, the L3 load was lower in BALB/c WT mice compared to those of C57BL/6 mice (Fig. 4a, compare to Fig. 1c). In addition, eosinophil frequencies were lower in cells isolated from the lungs of BALB/c wild type mice compared to C57BL/6 mice, but higher than in CBA mice at day 8 p.i. (compare Fig. 4b and Fig. 2c). Hence, the gradual eosinophil responses seen in the three wild type mouse strains appeared to result from the different parasite numbers rather than causing or contributing to the unequal L3 burdens.

In accordance, the lung sections of BALB/c wild type mice comprised intermediate numbers of F4/80^+^ macrophages/monocytes expressing Arg-1 at day 8 p.i. (Fig. 4c and supplementary Fig. 2b) compared to C57BL/6 and CBA mice (Fig. 3e). Histological lung sections of both BALB/c WT and dblGATA-1 mice indicated a modest rise in Arg-1^+^ cells, while, numbers of F4/80^+^ macrophages and Arg-1^+^ M2 macrophages/monocytes were significantly increased at day 8 p.i. in wild type animals (supplementary Fig. 2a, b). This indicates, that eosinophil infiltration observed in the lungs of BALB/c as well as in the lungs of C57BL/6 wild type mice (Fig. 4b and 2c) potentially reinforced macrophage/monocyte recruitment and pulmonary M2 polarization.

The intermediate eosinophilic and M2 macrophage response presented by BALB/c wild type mice at day 8 p.i. were accompanied by intermediate expression of IL-5/IL-13 by ILC2 and CD4 T cells (Fig. 4d). This moderately Th2-bound phenotype was mirrored in eosinophil-deficient dblGATA-1 mice, presenting a comparable increase in IL-5/IL-13 expression (Fig. 4d). Finally, both wild type and dblGATA-1 BALB/c mice displayed intermediate histopathological consequences of *Ascaris* infection in liver and lung, suggesting that eosinophils were not the main drivers of tissue inflammation and damage occurring within the acute stage of larval infection (Fig. 4e,f).

In summary (Fig. 4g), our data demonstrate that *A. suum* infection triggers eosinophil recruitment to the liver and lungs in susceptible C57BL/6 mice during larval migration. Further, an elevated type 2 cytokine production in the lung damaged by intruding larvae promoted the accumulation of M2 macrophages within the pulmonary tissue. Of note, M2 macrophages appeared to be mainly derived from recently recruited monocyte-derived CD11b^+^F4/80^+^Siglec-F^-^ macrophages, whereas alveolar Siglec-F^+^ macrophages did not express arginase 1 upon infection, but rather declined in frequencies over the course of infection. Interestingly, BALB/c mice displayed intermediate resistance to the lung stage of *Ascaris* which was accompanied by moderate eosinophil, M2 and Th2 cytokine responses as well as intermediate histopathology compared to C57BL/6 and CBA mice. None of the markers characteristic for a type 2 immune response was detectable at significantly elevated levels after infection in the resistant CBA strain, indicating that CBA mice control larval migration independent of type 2 immune circuits. Consequently, CBA mice largely lacked signs of pulmonary inflammatory cell infiltration and were thereby spared from type-2-related immunopathology. Furthermore, according to our results, eosinophils are not essential for protection against *Ascaris* larval migration during primary infection in the murine model, since similar larval burdens were seen in eosinophil-deficient mice. Hence, *A. suum* larval tissue-migration leads to initiation of type 2 responses in highly susceptible mice, however, this is associated with pathology rather than protection.

## Discussion

Humans and animals differ in their predisposition to helminth infections, and it is well documented that heavy infections lead to morbidity and eventually mortality when left untreated^7^. Nevertheless, the genetic and environmental traits rendering an individual susceptible or resistant to infection are poorly defined^33–35^. The importance of type 2 immunity and associated epithelial responses for the control of worm infections has been proven in a plethora of studies using rodent models of experimental helminth infections^36,37^. However, strong type 2 immunity is far less commonly seen in response to natural infections with parasitic nematodes in humans^38^, life stock^39–41^ and companion animals^42^, as well as in wild animals^43^. While the limited type 2 responses seen in natural worm infections may provide an explanation for the frequent reinfections of many helminth species in humans^44,45^ and other mammals^10^, the question of which factors influence the predisposition to severe worm infection remains open. The differential success in early larval migration seen in genetically distinct mouse strains provides a suitable model in which the relationship between immune responses and the intensity of *Ascaris* infection can be investigated^23^. Although this model allows to investigate the immune and tissue responses to the migration of *Ascaris* larvae affecting the lower small intestine and cecum, the liver parenchyma, and, subsequently, the lung tissue^16,17,22,24^, information on potential phenotypical differences in the adaptive and innate responses associated with the varying resistance of the different mouse strains is rather limited^46^. Therefore, we utilized this mouse model to comparatively investigate the efficacy of early type 2 immune responses in controlling body-migrating helminth larvae during primary *Ascaris* infection.

Consistent with previous findings^17,22,23,47^, our study confirmed the increased susceptibility of the C57BL/6 strain, as significantly more larvae migrated from the liver to the lungs than in the resistant CBA mouse strain. In contrast to another study showing equal larval loads in liver tissue at day 4 p.i.^22^, we already found fewer larvae in CBA mice at this time point, indicating slight differences in the kinetics of larval migration^24^ which may relate to factors such as mouse age, sex, origin and microbial colonization patterns between the studies. To exclude strain-specific differences in the initial intestinal invasion, we determined the number of eggs excreted in the feces within the first 6 h after oral inoculation. Importantly, we were able to show that hatching of embryonated *Ascaris* eggs in the intestine did not differ between the two mouse strains, confirming previous studies reporting no difference in the number of free larvae in the contents of the large intestine at 6 h p.i.^22,24^. The differences in hepatic and pulmonary larval load were therefore apparently not due to unequal efficiency in the invasion of the intestinal barrier by *Ascaris* L3, but likely due to differences in migration behavior, whereby substrain-specific differences (CBA/Ca in previous studies vs. CBA/JR in our study) could be relevant here. A recent comparative study in *Ascaris*-infected BALB/c and C57BL/6J mice showed a correlation between higher concentrations of total sIgA in the intestinal fluid and a lower parasite load in the more resistant BALB/c mice^48^. Thus, a comparison of the sIgA levels also for CBA and C57BL/6 mice might represent a valuable addition for future studies examining the intrinsic resistance mechanisms to larval ascariasis.

In the liver of both mouse strains, we also determined the degree of inflammation as a correlate for the extent of larval migration. Interestingly, despite the observed difference in liver larval load found between susceptible C57BL/6 mice and resistant CBA mice at day 4 p.i., less inflammation was present in the resistant CBA mice during liver and lung stage of infection. This is in line with the results of Dold et al. (2009), who showed that CBA mice are able to cope with comparable *Ascaris* liver migration more efficiently compared to C57BL/6 mice by allowing faster tissue recovery without additional inflammation^24^. Hence, the observed differences in liver larval load and histopathology in this study seem to relate to a potentially quicker and ‘immunologically silent’ hepatic trapping and elimination of *Ascaris* larvae by resistant CBA mice compared to susceptible C57BL/6 mice.

In fact, as a confirmation of the gradual increase in liver inflammation/damage occurring in hepatic tissue of susceptible mice during liver and lung stage of *Ascaris* infection, activation of Th2 responses as well as recruitment of inflammatory cells such as eosinophils and monocyte-derived macrophages were found significantly elevated at day 8 p.i. compared to resistant mice. Monocyte-derived macrophages have been shown to provide protection against other helminth infection in the liver by acquiring an alternative activated M2 macrophage phenotype^49^. However, polarization of these recently recruited cells and Kupffer cells, the tissue resident macrophages of the liver, towards the M2 phenotype was found to be not significantly different between the 2 mice strains in this study, suggesting *Ascaris*-mediated impairment of macrophage polarization during hepatic migration. Therefore, the increased inflammation observed in hepatic tissue of susceptible mice seem to be a consequential, but pathological rather than protective response associated to the incapability of this mouse strain to cope efficiently with *Ascaris* larvae during liver stage of infection.

Similarly, during lung stage of infection, we observed, due to the very low numbers of *Ascaris* larvae found in the lungs of resistant CBA mice, low or no signs of pulmonary inflammation compared the significantly increased pathology observed in lungs of susceptible C57BL/6 mice. Lewis et al.^22^ reported a similar finding in C57BL/6 mice at day 8 p.i. during *Ascaris* primary infection, where pulmonary inflammation was correlated with the increased infiltration of neutrophils, lymphocytes and eosinophils to the infected tissue^22^. Also, Oliveira et al.^50^ showing between day 7 until day 42 significant lesions and inflammation in the lung following primary *Ascaris* infection of C57BL/6 mice due to *A. suum* larval antigen fragments^50^. Likewise, we found increased frequencies of innate and adaptive lymphocytes that expressed IL-5^+^/IL-13^+^ as well as eosinophils. However, as a result of the increased innate and adaptive type 2 cytokine responses seen in lungs of susceptible mice, we were also able to determine the degree of lung M2 macrophage polarization linked to *Ascaris* primary pulmonary migration in susceptible and resistant mice.

The macrophage assessment showed no altered macrophage phenotype between naïve and infected CBA mice while high proportions of non-tissue resident macrophages were polarized towards the M2 phenotype marked by arginase 1 (Arg-1) and CD206 expression in lungs of susceptible mice at day 8 p.i.. These data are in line with Oliveira et al. 2024 who demonstrated a polarisation of lung macrophages towards the M2 phenotype via gene expression analysis of the M2 markers (MRC1, MMP12, IRF4)^50^. On the other hand, alveolar macrophages, the tissue resident macrophages of the lung, did not contribute significantly to the M2 macrophage pool, despite the increased frequencies of ILC2 and Th2 in the lungs of susceptible mice, as their frequencies gradually decreased at day 4 and 8 p.i. These results are consistent with previous studies that have shown that monocyte-derived macrophages replace alveolar macrophages and acquire an alternatively activated macrophage transcriptional profile induced by tissue-migrating nematodes, which persists long after worm elimination^51^. Hence, in susceptible mice, the increased *Ascaris* L3 count found in the lungs during primary infection seems to directly translates into an elevated number of recently recruited monocyte-derived M2 macrophages that present dim expression of F4/80^52^.

In addition, with regards to macrophage phenotype presented by BALB/c mice, we found interesting that although the number of M2 macrophages was significantly increased in the lungs of BALB/c wild-type mice compared to BALB/c dblGATA-1 mice, this was not related to protection as there was no difference in larval load and pathology in the lungs. Accordingly, a study by Nogueira et al.^21^ showed that the total number of macrophages in the bronchoalveolar lavage (BAL) of GATA1-/- mice was significantly lower than that of their wild-type counterparts with a comparable parasitic larval load in the BAL^21^. These findings are consistent with those reported by Bowcutt et al., (2011) during infection with the large intestinal nematode *Trichuris muris,* showing that helminth expulsion from C57BL/6 mice does not differ in the presence or absence of macrophage arginase 1 activity^53^. Hence, development of type 2 responses in *Ascaris* infected mice seems to not be related to an efficient resolution of infection-induced pathology and parasite clearance. However, our data do not exclude the possibility that the strong type 2 responses seen in C57BL/6 mice may eventually be suited for the control of the *Ascaris* larval stages and pathology during reinfection or even more importantly for the control of adult worms during chronic phase of *Ascaris* infection in natural hosts. Finlay et al.^54^ recently reported that Th2 cells induced in C57BL/6 during *Litomosoides sigmodontis* infection of the pleural cavity are needed for the tissue-resident macrophage dependent parasite control^54^. However, both CBA and, to a lesser extent, BALB/c mice were able to control larval migration more efficiently and, as a result, weaker type 2 responses were instructed. In this context, Nogueira et al.^21^ found that protein levels of IL-5 and IL-13 were significantly increased in the lung tissue of GATA1-/- mice compared to wild-type BALB/c mice, thereby confirming the findings of our study that early type 2 immune responses are dispensable for impeding larval migration, at least in the primary infection of the host^21^.

Eosinophil infiltration occasionally occurs as part of a protective type 2 innate immune response against a nematode infection^30^. Depending on the target (i.e. *Strongyloides ratti, Brugia pahangi and Brugia malayi*), these cells may affect larval stages by degranulation^55^, extracellular trap formation^26,56^ or antibody-dependent cytotoxicity^57^. However, studies investigating models such as *Heligmosomoides polygyrus, Schistosoma mansoni* and *Trichinella spiralis* showed that eosinophils are likewise dispensable for the control of other types of nematodes^25,58,59^. In the current study, we also detected a prominent infiltration of eosinophils to the liver and lungs, but also a significant accumulation in the peritoneal cavity and spleen during lung stage of infection. However, this did not negatively affect hepatic and pulmonary *A. suum* larval load in susceptible C57BL/6 mice, indicating that eosinophil infiltration and effector functions are dispensable for protection during *A. suum* larval migration in the primary infected host.

In fact, by using BALB/c dblGATA-1 mice, which are deficient in eosinophils, we were also able to demonstrate that eosinophils are not essential for protection against primary *Ascaris* infection. Independent of eosinophil presence, similar numbers of *Ascaris* larvae were detected in the lungs of infected GATA-1^-/-^ knock out and wild type mice. The results of our study are consistent with those of Nogueira et al.^21^, who showed that the total parasite load in the bronchoalveolar lavage (BAL) of dblGATA-1 mice remained unchanged compared to wild-type BALB/c mice^21^. However, the total parasite burden, including BAL and lung parenchyma, was significantly increased in their study. This is a limitation of our study as we did not additionally determine the larval load in the lung tissue. Nogueira et al.^21^ also demonstrated that the control of parasite load during the pulmonary phase was associated with Toll-like receptor (TLR) 2 and TLR4 signaling, which induced eosinophils and subsequently promoted secretory immunoglobulin A (SIgA) production^21^. However, 2 weeks of cohousing of the mice in our study before infection exclude major differences in bacterial stimuli to be responsible for the detected differences in the two studied mouse strains. Cadman et al.^60^, showed that eosinophil recruitment is related to nematode-induced pathology and impaired lung function in filarial infection^60^. Linked to this, the release of granule proteins, such as eosinophil peroxidase (EPO) and major basic protein-1 (MBP-1) results in alteration of lung physiology. In accordance, we found EPO to be significantly increased in serum of susceptible C57BL/6 mice compared to resistant CBA mice during lung stage of infection, and conclude that eosinophils recruited to the liver and lungs of susceptible mice promote tissue pathology rather than protection in *Ascaris*-infected mice.

In conclusion, although we performed a comprehensive phenotypic Th2 analysis of innate and adaptive immune cells, we were unable to decipher the cellular or molecular immune factors conferring protection to CBA mice in primary infection. Contrary to expectations, our data suggest that the rapid development of type 2 immune responses in C57BL/6 mice is not necessarily associated with resistance, as this mouse strain is significantly more susceptible compared to CBA mice, with the latter largely lacking signs of early type 2 responses during the initial phase of *Ascaris* infection. Rather, this strong systemic and local activation of type 2 immune responses correlates with increased tissue damage as a result of enhanced larval migration. It is known that resistance to infection with the tissue-migratory helminth *Nippostrongylus brasiliensis* is closely linked to the development of a Th2 immune response^61^. This is apparently not the case during primary *Ascaris* larval migration in the murine model, so that other factors must contribute to promoting resistance during primary infection, which require further investigation. Moreover, in the liver the larval loads of both strains are clearly detectable, die-off of *Ascaris* larvae in the resistant strain seems to happen in the time window before reaching the lung during the hepato-tracheal migration. This time window will be addressed in further detail in future studies.

## Materials and methods

### Animals and infection

For at least 2 weeks, under specific pathogen-free (SPF) conditions, 7 to 8 weeks old female wild-type CBA and C57BL/6 mice from Janvier Labs (Saint-Berthevin, France) were co-housed in individually ventilated cages. Under the same conditions 7 to 8 weeks old female wild-type BALB/c (BALB/c WT) and BALB/c eosinophil-deficient mice (BALB/c ΔdblGATA) were caged together upon arrival. Thereafter, 2,500 fully embryonated *Ascaris suum* eggs (preparation according to Kundik et al.^62^) suspended in 150 µL of drinking water were used to infect the 9-weeks old female mice via oral gavage. Naïve mice as well as mice at day 4 p.i. and day 8 p.i. were anesthetized with xylazine and ketamine and sacrificed by cervical dislocation. All the experiments followed the National Animal Protection Guidelines and were approved by the German Animal Ethics Committee for the Protection of Animals (LAGeSo, G0129/18).

### Egg count

6 h post infection, fecal material from mice cages was recovered. Mice feces were dissolved in tap water and filtrated using a 100 µm sieve. Filtered material was then resuspended in saturated NaCl solution and centrifuged to recover floating embryonated eggs. Recovered eggs were washed and counted using a light microscope on the same day of fecal material collection.

### Larval load

At day 4 p.i., livers from *A.* suum infected CBA and C57BL/6 mice were individually cut into small pieces and incubated overnight at 37 °C in a 6-well plate containing 5 mL of PBS supplemented with 1.5% Penicillin/Streptomycin. The *A.* suum larvae that emigrated from liver tissue and the pores of a 40 µm cell strainer that contained the minced mouse liver tissue were then enumerated using a light microscope. Similarly, at day 8 p.i. larvae were enumerated in bronchoalveolar lavage fluid by using a light microscope on the same day of dissection. Accordingly, the entire volume of bronchoalveolar lavage fluid was employed for the enumeration of larvae via microscopic examination.

### Cell isolation

Spleen and peritoneal cavity (PEC) single cell suspensions were obtained as described previously by Steinfelder et al.,^63^. Bronchoalveolar lavage (BAL) cells were obtained by flushing 5 times 800 µL of FACS buffer (PBS; PAN-Biotech with 0.2% BSA, 2 mM EDTA) into the lungs of mice via intratracheal canalization after euthanasia. To obtain single cell suspensions of liver and lung tissues, 20 mL of cold PBS were used to perfuse both organs via right heart ventricle injection. Thereafter, removed livers and lungs were digested using liver or lung dissociation kits from Myltenyi Biotec (Bergisch Gladbach, Germany) according to manufacturer’s instructions. Obtained cell suspensions were used for further flow cytometry analysis.

### In vitro stimulation

For the analysis of cytokines expressed by innate lymphoid cells (ILC) and CD4^+^ T cells, 2 million cells per well of whole liver, lung, PEC and spleen cells were plated out in a V-bottom 96-well cell culture plate (Greiner Bio-One, Germany). Using 200 µL of RPMI medium (PAN-Biotech), containing 10% FCS, 100 U/mL penicillin, and 100 µg/mL streptomycin (all from PAA, Pasching, Austria), cells were stimulated with phorbol-12-myristate-13-acetate (PMA) and Ionomycin at a concentration of 1 µg/mL for 1 h. Brefeldin A was then added in a 1000-fold dilution and cells were kept under stimulating conditions at 37 °C for 3 to 5 extra hours, before continuing with extracellular and intracellular staining of the stimulated cells.

### Flow cytometry

The antibodies used for flow cytometry are described in Table S1. To discriminate between death and alive cells eF506 fixable viability dye (Thermo Fisher, Waltham, USA) was used. For the intracellular staining, cells were fixed and permeabilized using the Fixation/Permeabilization kit and Permeabilization buffer (ThermoFisher/eBioscience). Samples were acquired on a FACSAria^™^ III (BD Biosciences, Heidelberg, Germany) and data analyzed using FlowJo version 10 (Tree star Inc., Ashland, OR, USA).

### Histopathology

A representative piece (approximately 0.5 cm^3^) of liver and lung tissue was formalin-fixed and embedded in paraffin. Paraffin sections were cut (1–2 µm thickness), dewaxed and rehydrated prior to histochemical staining using hematoxylin and eosin for overview or sirius red. Modified sirius red staining was performed for detection of eosinophil granulocytes^25^. Liver and lung inflammation were evaluated using H&E-stained sections and a AxioImager Z1 microscope (Carl Zeiss MicroImaging, Inc.). Liver inflammation was scored according to Siegmund et al.^64^ and lung inflammation according to Al-Shami et al*.*^39^. Eosinophils were quantified in 5 high power fields per section using the AxioImager Z1 microscope. For quantification of arginase 1-expressing macrophages, tissue sections were dewaxed and rehydrated in a descending alcohol series. After blocking endogenous peroxidase, sections were incubated with anti-arginase 1 (clone N20, 1:800; Santa Cruz) followed by incubation with rabbit anti-goat (1:1,000; Invitrogen). For visualization, the EnVision + System-HRP Labelled Polymer Anti-Rabbit (Dako) and OPAL-570 (Akoya Biosciences) was used. Subsequently, sections were subjected to heat-induced epitope retrieval and incubated with anti-F4/80 (clone D2S9R, 1:3,000; Cell Signaling Technologies). For visualization, the EnVision + System-HRP Labelled Polymer Anti-Rabbit (Dako) and OPAL-620 (Akoya Biosciences) was used and nuclei were stained using 4′,6-diamidine-2′-phenylindole dihydrochloride (DAPI; Sigma). All incubation steps were carried out at room temperature for 30 min. Slides were coverslipped in Fluoromount G (Southern Biotech) and multispectral images were acquired using a Vectra^®^ 3 imaging system (Akoya Biosciences). Positive cells were quantified in 5 high power fields (field of vision in × 200 original magnification) employing the inForm software (version 2.4.8) for spectral unmixing and cell segmentation as well as cell phenotyping. The cell phenotypes were quantified using RStudio (version 1.3.1073). Images show pseudocolours with DAPI (white), arginase 1 (red) and F4/80 (blue). All microscopic evaluations were performed in a blinded manner.

### Eosinophil peroxidase quantification in serum

Eosinophil peroxidase (EPO) levels in blood serum were quantified using the mouse eosinophil peroxidase (EPX) ELISA kit according to the manufacturer’s instructions (DLdevelop, Jiangsu, PRC). Serum samples were diluted 1:5 in diluent buffer and added to the plate with serially diluted standards. Absorbance was measured on a Biotek Synergy H1 Hybrid Reader at a wavelength of 450 nm. Data presented for this assay are pooled from three independent experiments with 3 mice per group in each experiment.

### Statistical analysis

T test was used to compare the means of groups of normally distributed data. On the other hand, Mann–Whitney test or Kruskal–Wallis test together with Dunn’s multiple comparison test were used to compare ranks of groups of non-normally distributed data. Normality of data was assessed by D’Agostino & Pearson test or Shapiro–Wilk test. Outliers were excluded using ROUT method. In brief, since 68.27% of values in a Gaussian distribution lie within one standard deviation of the mean, the scatter of points around the curve is calculated by robust linear regression taking into account the absolute values of the residuals of this percentile. The result value called Robust Standard Deviation of the Residuals (RSDR) is then used to approximate a two-tailed t distribution of P values calculated by dividing each residual by the RSDR. Finally, the False Discovery Rate method is used to determine which of these P values is ‘significant’, and define the corresponding points to be outliers. GraphPad Prism software version 9.0.1 (La Jolla, CA, USA) was used for all statistical analysis. In bar plots, the mean and standard deviation are shown. For simplicity, non-significant differences are not reported. P-values of < 0.05 were considered statistically significant.

### Ethical approval

All methods are reported in accordance with the ARRIVE guidelines for animal experiments.

### Supplementary Information

Supplementary Figures.

Supplementary Table S1.

## Data Availability

The data that support the findings of this study are available from the corresponding author upon reasonable request.
