# Supplementary material for: Th2-biased immune responses to body migrating Ascaris larvae in primary infection are associated with pathology but not protection
Source: Sci Rep. 2024 Jun 28;14:14919. doi: 10.1038/s41598-024-65281-0 (PMC11213949; doi:10.1038/s41598-024-65281-0)
Supplement: Supplementary file 1 — Supplementary Figures. [file 41598_2024_65281_MOESM1_ESM.pdf]

Supplementary Fig. 1.

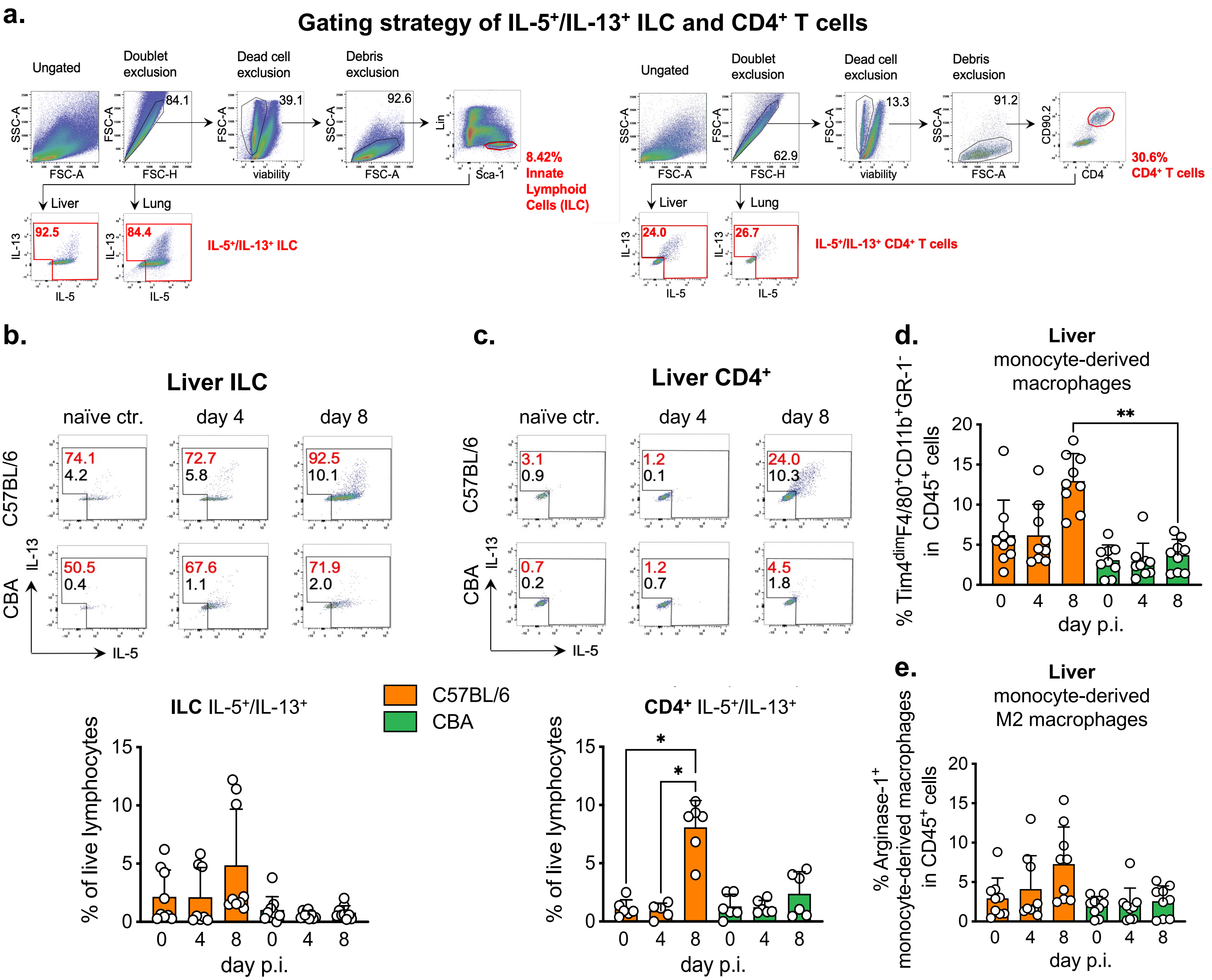

Supplementary Fig. 2.

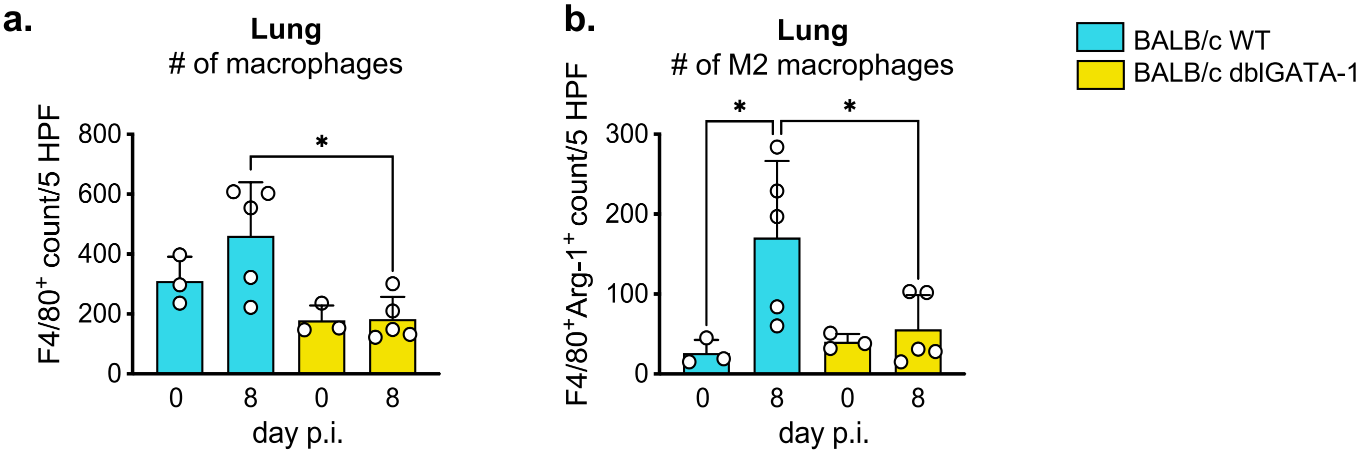

Supplementary figure 1. Liver type 2 immune responses presented by C57BL/6 mice and CBA mice. a) Gating strategies used to identify IL-5$^+$/IL-13$^+$ innate lymphoid cells (ILC) (Lin$^-$(CD3e$^-$CD11b$^-$CD45R/B220$^-$Ly-76$^-$Ly6G/Ly6C$^-$)Sca-1$^+$) and CD4$^+$ T cells (CD90.2$^+$CD4$^+$) in the liver and lung tissue, exemplarily shown for lung tissue of day 8 infected susceptible C57BL/6 mice. b, c) Frequencies of liver IL-5$^+$/IL-13$^+$ innate lymphoid cells (ILC) and CD4$^+$ T cells. The percentage of cytokine positive ILC and CD4$^+$ T cells is reported by red numbers. Black numbers report the frequencies of cytokine producing cells in live lymphocytes. d, e) Frequencies of liver monocyte-derived macrophages (Tim4^{dim}F4/80$^+$CD11b$^+$GR-1$^-$ and Arg-1$^+$ monocyte-derived macrophages. Pooled data from 2 to 3 independent experiments with n=3 to 6 animals per time point. $p < 0.05$ *; $p < 0.01$ **.

Supplementary figure 2. Numbers of macrophages and M2 macrophages presented in lung tissue by BALB/c wild type (WT) and BALB/c dblGATA-1 mice. a, b) Number of macrophages (F4/80$^+$) and arginase 1 positive macrophages (F4/80$^+$Arg-1$^+$) per 5 high power fields (HPF) in lung tissue. Data from 1 experiment with n=3 to 5 animals per time point. $p < 0.05$ *.
