# Supplementary material for: Th2-biased immune responses to body migrating Ascaris larvae in primary infection are associated with pathology but not protection
Source: Sci Rep. 2024 Jun 28;14:14919. doi: 10.1038/s41598-024-65281-0 (PMC11213949; doi:10.1038/s41598-024-65281-0)
Supplement: Supplementary file 2 — Supplementary Table S1. [file 41598_2024_65281_MOESM2_ESM.docx]

**Supplementary figures**

**Table S1. Fluorochrome, clone and company of monoclonal antibodies used for flow cytometry**

| **Antibody** | **Clone** | **Fluorochrome** | **Company** |
| --- | --- | --- | --- |
| CD11b | M1/70 | APC-Cy7 | BioLegend |
| CD11b | M1/70 | FITC | BD Bioscience |
| CD11b | M1/70 | BV605 | BioLegend |
| CD11b | M1/70 | A700 | Thermo Fisher Scientific |
| CD11c | HL3 | FITC | BD Bioscience |
| CD107a | 1D4B | A488 | BioLegend |
| CD127 | A7R34 | PE | BioLegend |
| CD19 | eBio1D3 | FITC | Thermo Fisher Scientific |
| CD206 | C068C2 | A647 | BioLegend |
| CD206 | C068C2 | PE-Cy7 | BioLegend |
| CD25 | PC61.5 | A700 | Thermo Fisher Scientific |
| CD25 | PC61 | PerCP-Cy5.5 | BD Bioscience |
| CD3 | 154-2C11 | FITC | Thermo Fisher Scientific |
| CD335 | 29A1.4 | A647 | BioLegend |
| CD4 | RM4-5 | A700 | BD Bioscience |
| CD45 | 30-F11 | A700 | BioLegend |
| CD49b | DX5 | PE | Thermo Fisher Scientific |
| CD5 | 53-7.3 | Biotin | Thermo Fisher Scientific |
| CD62-L | MEL-14 | APC-ef780 | Thermo Fisher Scientific |
| CD69 | H1.2F3 | PE-Cy7 | Thermo Fisher Scientific |
| CD8 | 53-6.7 | BV605 | BD Bioscience |
| CD80 | 16-10A1 | BV605 | BioLegend |
| CD90.2 | 30-H12 | PerCP-Cy5.5 | BioLegend |
| Arg1 | A1exF5 | APC | Thermo Fisher Scientific |
| Arg1 | A1exF5 | PE | Thermo Fisher Scientific |
| F4/80 | BM8 | ef450 | Thermo Fisher Scientific |
| GATA-3 | TWAJ | ef660 | Thermo Fisher Scientific |
| GR-1 | RB6-8C5 | APC-Cy7 | BioLegend |
| GR-1 | RB6-8c5 | PE-Cy7 | Thermo Fisher Scientific |
| IFNγ | XMG1.2 | ef450 | Thermo Fisher Scientific |
| IL-4 | 11B11 | PE-Cy7 | Thermo Fisher Scientific |
| IL-4 | 11B11 | BV605 | BioLegend |
| IL-5 | TRFK5 | PE | Thermo Fisher Scientific |
| IL-6 | MP5-20F3 | ef450 | Thermo Fisher Scientific |
| IL-13 | eBio13A | APC-ef780 | Thermo Fisher Scientific |
| IL-13 | eBio13A | A488 | Thermo Fisher Scientific |
| iNOS | CXNFT | PE-ef610 | Thermo Fisher Scientific |
| Ki67 | SoIA15 | PE-Cy7 | Thermo Fisher Scientific |
| Lin | 145-2C11, M1/70, RA3-6B2, TER-119, RB6-8C5 | APC | BD Bioscience |
| I-A / I-E | M5/114.15.2 | PerCP-Cy5.5 | BD Bioscience |
| Sca-1 | D7 | PE-Cy7 | Thermo Fisher Scientific |
| Siglec-F | E50-2440 | BB515 | BD Bioscience |
| ST-2 | RMST2-2 | Biotin | mdbioproducts |
| TCRβ | H57-597 | Biotin | BD Bioscience |
| TCRγ/δ | eBioGL3 | FITC | Thermo Fisher Scientific |
| Tim4 | RMT4-54 | PE | BioLegend |
| Streptavidin | --- | ef450 | Thermo Fisher Scientific |
| Streptavidin | --- | FITC | Thermo Fisher Scientific |
| Streptavidin | --- | APC-ef780 | Thermo Fisher Scientific |
| Streptavidin | --- | PerCP-Cy5.5 | BioLegend |
